# Early school closures can reduce the first-wave of the COVID-19 pandemic development

**DOI:** 10.1007/s10389-020-01391-z

**Published:** 2020-10-15

**Authors:** Monika Klimek-Tulwin, Tytus Tulwin

**Affiliations:** 1 Department of Laboratory Diagnostics, Medical University of Lublin, Lublin, Poland; 2 Department of Thermodynamics, Fluid Mechanics and Aircraft Propulsion, Lublin University of Technology, Lublin, Poland

**Keywords:** COVID-19, SARS-CoV-2, Pneumonia, Virus diseases, 2019 novel coronavirus infection, Epidemics

## Abstract

**Aim:**

The COVID-19 pandemic presents serious threats to global public health and the world economy. Therefore, the rapid escalation of the number of cases has led to national government and global interventions. This study aimed to assess the effect of school closures on the COVID-19 pandemic and epidemic trajectories in selected countries.

**Subject and methods:**

Information on the number of cases and population in each country were taken from official government reports. Dates of educational institutions closure were taken from the UNESCO database. Statistical analyses were performed using Statistica. We summarized the data graphically and descriptively.

**Results:**

Most of the European countries closed schools in the period of 11–20 of March 2020. However, there was a big difference in the phase of the epidemic on the day of closure. The data indicate that there was a strong correlation between the day of educational facilities closure and the incidence rate in the following days (16th, 30th, and 60th days since the 100th confirmed case in each country). Early closure of schools in analyzed countries is statistically significantly correlated with lower incidence rates further on during the different phases of the epidemic. Thereby closure of schools with delay is statistically significantly correlated with a higher incidence rate in the following days.

**Conclusion:**

The available data suggest that school closures can potentially reduce transmission during the pandemic, although more research is needed on the effectiveness of these practices.

## Background

In December 2019, a novel coronavirus was detected in three patients with pneumonia in Wuhan, the capital of Hubei province in central China. The virus is currently known as Severe Acute Respiratory Syndrome Coronavirus 2 (SARS-CoV-2) and the disease it causes is called COVID-19. After the COVID-19 outbreak in China, the epidemic further spread geographically. By the end of February 2020, several countries, including those in Europe, detected local transmission of the novel coronavirus SARS-CoV-2 (European Centre for Disease Prevention and Control (ECDC) 2020). On March 11, 2020, the Director-General of the World Health Organization (WHO) declared the COVID-19 pandemic (World Health Organization 2020). The COVID-19 pandemic presents serious threats to global public health and the economy. Therefore, the rapid escalation of the number of cases has led to national government and global interventions, such as closing public spaces, banning travel, and quarantining. These often infringe on individual rights, freedoms, and disrupt the regular activity in the interests of limiting the spread of the epidemic. Even though we need to take the infection seriously, these responses may contribute to social unrest, over-reaction, fear, and panic (Loveday 2020) (Abbasi 2020). Most governments instituted a temporary closure of national educational institutions in an attempt to contain the spread of the COVID-19 pandemic. These nationwide closures impacted over 91% of the world’s student population (UNESCO Institute for Statistics (UIS) database 2020). This paper reflects on the impact of school closure on COVID-19 epidemic trajectories in selected countries to establish its effect on transmission.

## Methods

Data collection. Information on the number of cases and population in each country is taken from official government reports collected by Worldometers.info (Worldometers.info 2020). The dates of national educational institution closure have been taken from the UNESCO Institute for Statistics database (UNESCO Institute for Statistics (UIS) database 2020).

Data analysis. Statistical analyses were performed using Statistica (Software TIBCO Software Inc. (2017). Statistica (data analysis software system), version 13. http://statistica.io.). The Shapiro–Wilk W test was applied to assess normal data distribution. A non-parametric Wilcoxon test was performed to compare data from each time-point. We summarized the data graphically and descriptively.

## Results

Fifteen countries from different epidemiological subregions, according to WHO-CHOICE, were included in the analysis. The WHO-CHOICE project divides the world into 14 subregions that have been grouped together based on geographical location, epidemiological profiles, costs, the effectiveness of health interventions, infrastructure, and economic situation (Table 1) (Tan-Torres et al. 2003).

**Table 1:** Analyzed countries divided into subregions

Abbreviation	Subregions	Countries
AMR	B	Argentina, Brazil
EUR	A	Belgium, Czechia, Finland, France, Germany, Italy, Norway, Spain, UK
EUR	B	Poland, Romania
EUR	C	Estonia, Hungary, Latvia, Lithuania
WPR	A	Japan

AMR = Americas, EUR = Europe, WPR = West Pacific, A = very low rates of adult and child mortality, B = low adult mortality, low child mortality, C = high adult mortality, low child mortality

**Fig. 1:**
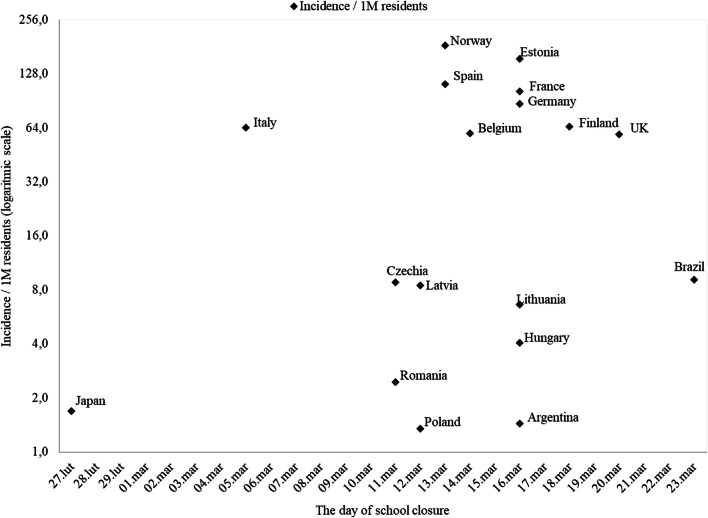
The incidence per 1 million residents (marker) on the date of educational facilities closure

Most of the European countries closed schools in the period of 11–20 of March 2020. Despite that, there is a big difference in the incidence rate on the day of closure among analyzed countries. Most of EURO A countries (Belgium, Finland, France, Germany, Italy, Norway, Spain, UK) closed schools when the incidence was close to or higher than 60 per 1 million residents (1 M). In contrast, countries from subregions EURO B (Poland, Romania), EURO C (Hungary, Latvia, Lithuania), and AMRO B (Argentina, Brazil) closed schools when the incidence rate was below 10/1 M (Fig. 1) (Table 2).

**Table 2:** The characteristics of incidence rate on the day of school closure in analyzed countries divided into subregions

Incidence/1 M on the day of schools closure						
Subregions	No.	Average	Median	Minimum	Maximum	SD
A	10	74,2	64,3	1,7	184,1	52,3
B	4	3,6	1,9	1,3	9,1	3,7
C	4	43,4	7,5	4,0	154,6	74,1

A = very low rates of adult and child mortality, B = low adult mortality, low child mortality, C = high adult mortality, low child mortality

**Fig. 2:**
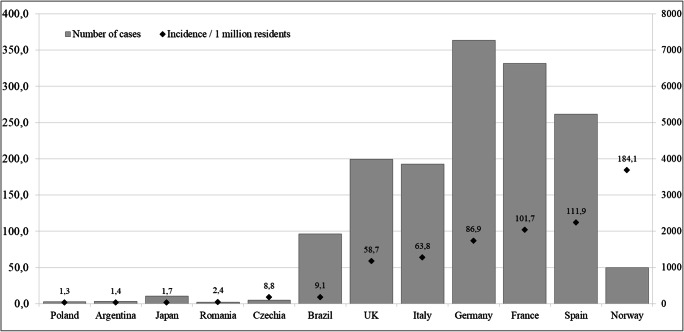
The number of cases (column) and the incidence per 1 million residences (marker) on the day of educational facilities closure

Due to significant differences in population size in analyzed countries, the number of confirmed cases is not an accurate indicator of the epidemic phase. Therefore, to compare the phase of the epidemic on the day of school closure, the incidence rate was used. As has been shown in Fig. 2, educational institutions were closed at an early stage of the epidemic’s development in Poland (1,3/1 M), Argentina (1,4/1 M), Japan (1,7/1 M), and Romania (2,4/1 M). By comparison, in Norway, Estonia, Spain, and France, this decision was taken with a significant delay when the spread of the infection was already much higher (>100/1 M) (Fig. 2).

**Fig. 3:**
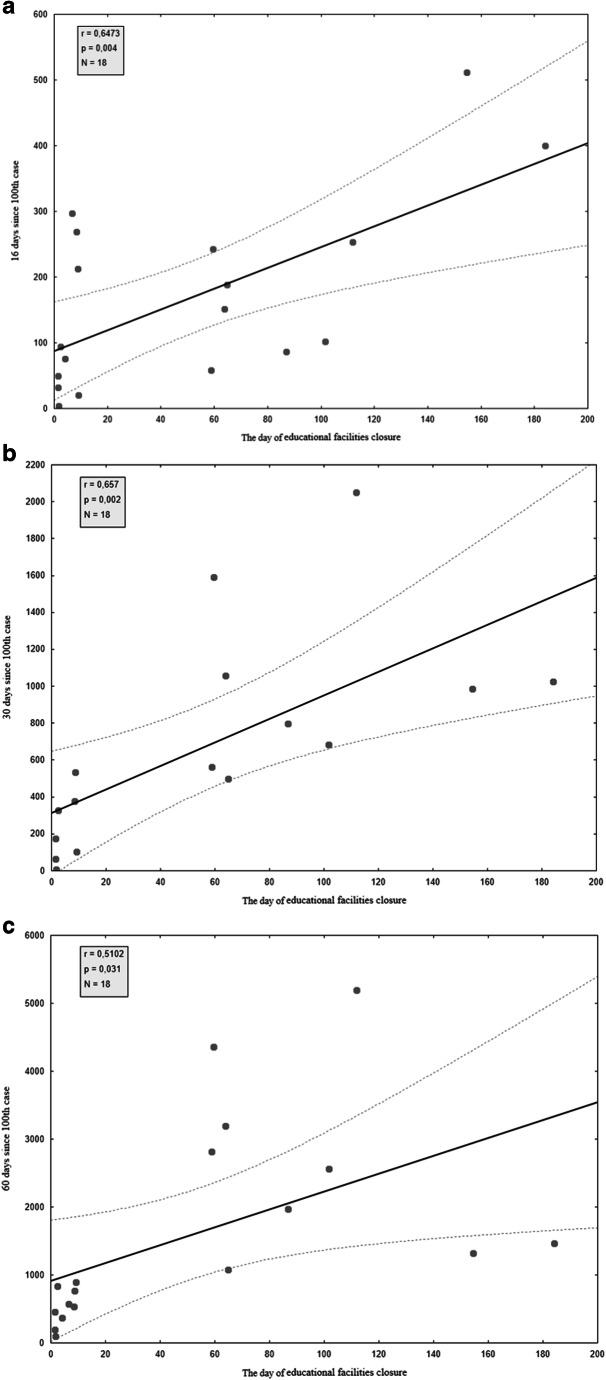
Correlation between the incidence/1 M in the date of educational facilities closure and the incidence/1 M in the following days since 100th case: (a) 16 days, (b) 30 days, (c) 60 days

Correlation between the incidence rate on the day of school closure and the incidence rate in the following days was calculated to assess the impact of this governmental intervention. To reduce the impact of different phases of the epidemic in the analyzed countries, we assessed the incidence rate on the 16th, 30th, and 60th days since the 100th confirmed case in each country. Therefore, we can assume that we compare the incidence rate at a similar stage of the epidemic in each country. Correlation between the date of school closure and the incidence/1 M population in the following days is shown in Fig. 3. Moreover, the Wilcoxon test was performed to determine the significance of differences between pairs of the results from different time-points. The data indicate that significant differences exist in all cases: educational institutions closure day & 16th day since 100th case (*p* = 0.004), closure day & 30th day since 100th case (*p* = 0.002), closure day & 60th day since 100th case (*p* = 0.031).

**Fig. 4:**
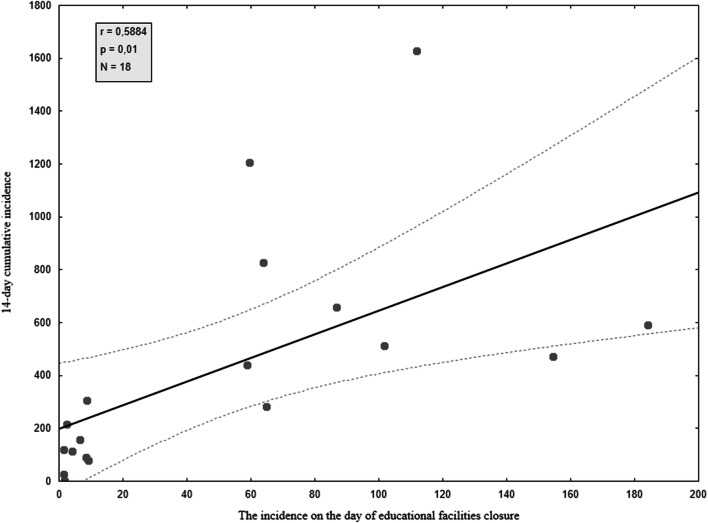
Correlation between the incidence/1 M population in the date of educational facilities closure and the 14-day cumulative incidence/1 M population

Correlation between the incidence rate in the date of educational facilities closure and the 14-day cumulative incidence rate at the early stage of the epidemic (from the 16th to the 29th day since the 100th case in each country) was also calculated. The 14-day cumulative incidence rate was calculated by dividing the total number of new cases over 14 days by the population of the country (Fig. 4).

There is a strong positive linear correlation between the day of educational facilities closure and the incidence rate in the following days. Early closure of schools in analyzed countries is statistically significantly correlated with lower incidence rates further on during the different stages of the epidemic. Thereby closure of schools with delay is statistically significantly correlated with a higher incidence rate in the following days. However, the correlation is stronger in the first month of the epidemic compared with the second month.

## Discussion

The available data suggest that school closures can potentially reduce transmission during the pandemic. In analyzed countries, earlier educational facilities closure was followed by a reduction in incidence in the general population. However, each country has its specificity as regards the development of an epidemic, and closure often occurred at a different phase in the outbreaks. Half of those analyzed closed schools before the high incidence rate, while the rest closed schools in a relatively late phase of the epidemic. Thus, the optimal school closure strategy is unclear.

School closures can affect an outbreak either positively, through reducing transmission and the number of cases, or negatively, through reductions in the healthcare workforce available to care for patients. School closures might lead to adults staying home, and consequently, on the other hand, many healthcare workers must reduce time spent providing patient care (Bayham and Fenichel 2020).

What we know regarding educational facilities closure effectiveness is based mainly on models of influenza, in which children are very susceptible to the disease. Systematic reviews of the effects of school closure on influenza outbreaks suggest that it can reduce the transmission of the pandemic. However, the empirical evidence did not resolve how or when to close schools. Furthermore, some authors suggest that a delayed introduction of non-pharmaceutical interventions, including school closures, may be associated with lower effectiveness of this intervention (often close to or after the peak) (Jackson et al. 2013).

Pediatric COVID-19 cases might be less severe, often asymptomatic, and children might experience different symptoms than adults. Therefore, social distancing and everyday preventive behaviors remain essential for all age groups because patients with less severe illness and those without symptoms likely play an essential role in disease transmission (CDC COVID-19 Response Team 2020). The school closure as an isolated measure was predicted to reduce coronavirus infections by around 5.6%. Recent modeling studies predicted that school closures would reduce the total deaths by around 2–4% during a COVID-19 outbreak. Currently, the evidence to support national closure of schools to combat COVID-19 is weak, and data from influenza outbreaks suggest that school closures could have relatively small effects on a virus with COVID-19’s high transmissibility and apparent low clinical effect on school children. If these results are confirmed, the benefits of transmission reduction from school closures will be even more reduced compared with those from influenza.

The long-term effects of closing schools are unclear, as relatively few studies presented substantial data after schools reopened. Some studies have concluded that reopening school after holiday periods can accelerate epidemic growth (Viner et al. 2020). Moreover, data show that school closures can have profound economic and social consequences. Therefore, the potential benefits of school closures as aimed at reducing asymptomatic virus transmission and the spread of infection should be balanced with their costs (Viner et al. 2020; Bayham and Fenichel 2020). The impact of other non-pharmaceutical interventions such as early border closure and isolation on the reduction of the COVID-19 epidemic has been widely described in other sources (Lai et al. 2020) (Lau et al. 2020) (Wilder-Smith and Freedman 2020).

Despite the introduction of radical restrictions, in most of the countries affected by the epidemic, the number of infected people shows an exponential trend. As a result, there is great concern regarding both mortality and the limited capacity of the healthcare system to respond effectively to the growing number of infected patients requiring intensive medical care. Reliable public health systems should have the resilience to address massive health threats with the collective responses they require. The actual capacity of the health care system during an epidemic will be affected not only by the number of cases and the daily increase in new cases but also by the number of public funds allocated to health care, the number of medical staff and hospital beds, equipment, premises, and logistics facilities. These factors may vary significantly between countries. Japan introduced appropriate containment measures and governance structures, supported health care delivery and financing, and developed and implemented plans and management structures. Japan relies on strong public health systems that enjoy full support and that can reach and mobilize the population. These also demonstrate that the trust of patients, health care professionals, and society as a whole in government are essential for meeting health crises (De Ceukelaire and Bodini 2020) (Legido-Quigley, et al., Legido-Quigley et al. 2020) (Abbasi 2020).

The limitation of this analysis is the fact that the total number of cases and the number of new cases may be underestimated due to the unstructured reporting by different countries. Moreover, frequent asymptomatic or minimally symptomatic courses of the disease result in some patients never being tested for COVID-19. An important factor influencing the number of confirmed cases is also the significant differences in a number of tests performed for SARS-Cov-2 among different countries. It depends, in particular, on the phase of the epidemic and the capacity of the healthcare system (Mizumoto et al. 2020).

## Conclusion

The available data suggest that school closures can potentially reduce transmission during the pandemic. However, strong evidence is not available for the effectiveness of these practices, and the optimal school closure strategy is unclear. More modeling and observational research are urgently needed on the effectiveness of school closures to inform policies related to COVID-19.
